# Long-term disability after initiation of platform versus high-efficacy disease-modifying therapy in relapsing-onset multiple sclerosis

**DOI:** 10.1007/s00415-026-13790-5

**Published:** 2026-04-01

**Authors:** Jie Guo, Tomas Olsson, Eva Johansson, Lars Alfredsson, Anna Karin Hedström

**Affiliations:** 1 Department of Nutrition and Health, China Agricultural University, Beijing, China; 2 Department of Clinical Neuroscience, Karolinska Institutet, Stockholm, Sweden; 3 Institute of Environmental Medicine, Karolinska Institutet, Stockholm, Sweden; 4 Centre for Occupational and Environmental Medicine, Region Stockholm, Stockholm, Sweden

**Keywords:** Multiple sclerosis, Relapse-associated worsening, Progression independent of relapse activity, Disease-modifying therapy, Expanded disability status scale

## Abstract

**Background:**

Several observational studies have compared high-efficacy and platform disease-modifying therapies (DMTs) with respect to long-term disability in relapsing-onset multiple sclerosis (MS), yet it remains unclear whether observed differences reflect relapse-associated worsening (RAW), progression independent of relapse activity (PIRA), or both.

**Methods:**

We included 2,563 DMT-naïve individuals with relapsing-onset MS enrolled in a population-based study linked to the Swedish MS registry (40 clinics, 2005–2019). The exposure was initial DMT efficacy class (platform versus high-efficacy therapy), with platform as the reference. Cox models estimated hazard ratios (HRs) with 95% confidence intervals (CIs) for RAW, PIRA, and time to EDSS 3 and 4. EDSS trajectories were modeled using mixed-effects models. Follow-up started at DMT initiation and was censored at treatment switch, discontinuation, death, drop-out, or study end.

**Results:**

At treatment initiation, 1,987 participants started a platform DMT and 576 a high-efficacy DMT. High-efficacy therapy was associated with a lower risk of RAW (HR 0.60, 95% CI 0.38–0.92), while the risk of PIRA did not differ between treatment groups (HR 1.05, 95% CI 0.79–1.39). Risks of reaching EDSS 3 and EDSS 4 were also lower with high-efficacy DMT (EDSS 3: HR 0.26, 95% CI 0.17–0.38; EDSS 4: HR 0.32, 95% CI 0.18–0.54). EDSS trajectories increased more steeply among platform-treated participants, with partial convergence toward the high-efficacy group over time.

**Conclusions:**

Our findings suggest that inflammatory and relapse-independent components of MS disability respond differently to current therapies and highlight the need for complementary neuroprotective strategies.

**Supplementary Information:**

The online version contains supplementary material available at 10.1007/s00415-026-13790-5.

## Introduction

Multiple sclerosis (MS) is a chronic inflammatory and neurodegenerative disease of the central nervous system that leads to accumulation of irreversible disability in a substantial proportion of patients. Disability is commonly quantified with the Expanded Disability Status Scale (EDSS) [1], which captures ambulation and neurological function and is used both in clinical practice and as a key outcome in trials and observational studies [2, 3]. Although relapses dominate early disease, disability may accrue independently of overt relapses through inflammatory and neuroaxonal processes [4–6]. Preventing long-term disability remains a primary therapeutic goal.

Over the past 2 decades, disease-modifying therapies (DMTs) have expanded from platform agents to high-efficacy therapies that more potently suppress disease activity. Randomized trials consistently show larger effects of high-efficacy DMTs on relapses and MRI activity than platform agents [7–9], yet their impact on long-term disability accumulation is less certain. Observational studies often suggest benefits from initiating or switching to high-efficacy therapy [10–13], but not all have detected between-treatment differences [14]. Heterogeneity likely reflects susceptibility to confounding by indication and informative censoring, and EDSS outcomes generally require extended follow-up to reveal durable effects.

Using a population-based, incident MS cohort with longitudinal follow-up, we compared long-term EDSS progression in DMT-naïve participants with relapsing-onset MS initiating platform versus high-efficacy DMTs. We examined relapse-associated worsening (RAW), progression independent of relapse activity (PIRA), time to EDSS milestones, and EDSS trajectories over extended follow-up. We hypothesized that high-efficacy therapy would be associated with more favorable long-term disability outcomes.

## Methods

Participants were drawn from the population-based case–control study Epidemiological Investigation of MS (EIMS) [15] involving the general Swedish population aged 16–70 years. Incident cases of MS were enrolled via 40 neurology units nationwide, including all university clinics. Cases were invited to participate in EIMS at diagnosis. Eligibility criteria for cases were age 16–70 years, residence in Sweden, and a neurologist-confirmed MS diagnosis according to the McDonald criteria applicable at the time of diagnosis [16].

Between April 2005 and April 2019, 3567 cases completed a standardized questionnaire collecting detailed information on environmental exposures and lifestyle factors (response rate 93%). We excluded participants with incomplete information on DMT in the Swedish MS register (*n* = 64), those who never initiated DMT (*n* = 291), those with progressive-onset MS (*n* = 171), and those without EDSS assessments while receiving their initial DMT, (*n* = 478), yielding a final analytic cohort of 2,563 participants with relapsing-onset MS.

### Standard protocol approvals, registrations, and patient consents

The study was approved by the Regional Ethical Review Board at Karolinska Institute (reference number 2004-252/1–4, 2013/1691–32 and 2025-00340-2) and conducted in accordance with the Declaration of Helsinki. All participants provided informed written consent.

### Definition of exposures and outcome measures

All participants were linked to the Swedish MS registry to obtain longitudinal clinical information [17]. The registry is embedded in nationwide routine neurological care and prospectively captures treatments, relapses, EDSS scores, and patient-reported outcomes entered by clinicians at follow-up visits. Relapse documentation includes clinical assessment with recorded dates, and reporting procedures are standardized across participating clinics.

Platform therapy included interferon-beta, glatiramer acetate, teriflunomide, and dimethyl fumarate. High-efficacy DMTs included natalizumab, fingolimod, ocrelizumab, alemtuzumab, cladribine, mitoxantrone, and rituximab.

Confirmed disability worsening (CDW) was defined as an EDSS increase from baseline sustained for at least 6 months (≥ 1.5 points if EDSS at baseline was 0, ≥ 1 point if baseline EDSS was 1.0–5.0, and ≥ 0.5 points if baseline EDSS was ≥ 5.5). Each CDW event was classified according to relapse activity around the time of EDSS increase. Events were classified as RAW if a clinical relapse occurred at any time between the most recent EDSS assessment preceding the CDW event and the EDSS increase defining CDW. Events not meeting this criterion were classified as PIRA. This definition was chosen to reflect the structure of longitudinal EDSS assessments in routine clinical practice. In this competing event framework, RAW and PIRA represent mutually exclusive types of CDW events. Time to EDSS milestones 3 and 4 was assessed among participants with a baseline EDSS below 3.

### Statistical analysis

#### DMT and time-to-event outcomes

Multivariable Cox proportional hazard regression was used to examine the association between DMT efficacy and RAW, PIRA, and time to EDSS 3 and EDSS 4. For analyses of RAW and PIRA, cause-specific hazard models were applied, treating the alternative type of CDW event as a competing event and censoring individuals at the time of the competing event. For EDSS milestone analyses, standard Cox proportional hazard models were applied without competing risk modeling of RAW and PIRA. Hazard ratios (HRs) with 95% confidence intervals (CI) were reported. Analyses were restricted to DMT-naïve participants initiating their first DMT. Follow-up began at treatment initiation and person-time accrued only while participants remained within their original efficacy category. Follow-up ended at treatment discontinuation, treatment switch across classes, outcome event, drop-out, death, or end of follow-up (April 6, 2022), whichever occurred first. The proportional hazard assumption was tested using Schoenfeld residuals and no violations were observed.

All analyses were adjusted for sex, calendar year of diagnosis, time between onset and treatment initiation, age at treatment initiation, and baseline EDSS defined as the EDSS measurement recorded closest to DMT initiation (mean time difference between DMT initiation and baseline EDSS was 0.44 years).

#### DMT and long-term disability trajectories

We modeled EDSS over time using mixed-effect models, intended as descriptive summaries of mean trajectories. First, EDSS trajectories were modeled, while participants remained within their original efficacy category, ensuring the analysis reflected sustained treatment effects.

We also examined patients who escalated from platform DMT to high-efficacy therapy.

For this subgroup, the switch defined time zero. EDSS was modeled over the 5 years before and 10 years after the switch. EDSS measurements after discontinuation of high-efficacy DMT among switchers and after cessation of platform DMT among non-switchers were not considered. Two distinct trajectories were plotted: one for participants who maintained platform DMT up to time zero and another for those who escalated at time zero.

#### Sensitivity analyses

To improve comparability between treatment groups and reduce heterogeneity related to disease stage and treatment initiation, we first repeated the primary analyses restricted to participants with a baseline EDSS < 3. This restriction was chosen to focus on individuals with mild disability at treatment start, where both platform and high-efficacy DMTs are commonly considered in routine clinical practice.

For comparability with prior studies, we additionally examined a narrower relapse ascertainment window defining RAW as relapse occurrence within 90 days prior to the EDSS increase. CDW events not meeting the relapse criteria were classified as PIRA. All other aspects of the analytical approach were unchanged.

In further sensitivity analyses, we extended covariate adjustment to include demographic and lifestyle factors, including ancestry (Nordic vs non-Nordic), educational attainment (pre-secondary, secondary, or post-secondary school), another autoimmune disease (yes vs. no), past infectious mononucleosis (yes, no, or unsure), smoking status (smokers vs non-smokers), alcohol use (yes vs. no), body mass index (categorized as < 25, 25–30, or > 30 kg/m^2^), fish consumption, sun exposure habits, and physical activity. Fish consumption was assessed by asking about the average consumption of lean and oily fish. Responses were recorded on a 4-point scale. We constructed a frequency score for fish consumption by summing the responses, yielding a value between 2 (lowest exposure) and 8 (highest exposure). Based on three questions regarding sun exposure at diagnosis (sunbathing in Sweden, traveling to sunnier countries, and use of sunbeds), where each answer alternative was given a number ranging from 1 (the lowest exposure) to 4 (the highest exposure), we constructed an index by summing the scores (range 3–12) and dichotomized at the median into high vs low exposure. Physical activity at diagnosis was dichotomized as regular (exercising for at least 30 min 1–2 times per week) and non-regular (low or moderate physical activity without sweating). To account for changes in diagnostic criteria and available therapies, we additionally performed a sensitivity analysis restricted to participants diagnosed in 2010 or later. Finally, to minimize potential immortal time bias, sensitivity analyses were also performed with start of follow-up defined as the date of the baseline EDSS measurement rather than DMT initiation.

To assess potential selection bias related to early treatment changes and missing EDSS assessments during the initial DMT course, we compared participants excluded due to missing EDSS (*n* = 478) with those included in the study (*n* = 2563). Because EDSS assessments are scheduled annually in routine care, missing EDSS after DMT initiation may reflect early treatment escalation rather than loss to follow-up.

As a conservative stress test, we repeated the analyses including all participants irrespective of EDSS availability. For participants who initiated platform therapy and subsequently escalated to high-efficacy DMT without a recorded EDSS during the initial course, escalation was assumed to reflect inflammatory disease activity and was treated as RAW. All other participants without an EDSS assessment were right censored at 12 months after DMT initiation (or earlier at treatment stop or study end). RAW and PIRA definitions were otherwise unchanged, and cause-specific Cox models were re-estimated on the augmented dataset. This analysis was intended as a stress test to explore the potential impact of informative exclusion of early switchers, acknowledging that escalation may also occur for reasons other than clinical relapses.

Finally, to address potential confounding by indication, we conducted a sensitivity analysis using inverse probability of treatment weighting (IPW) based on baseline propensity scores. The propensity score for initiation of platform vs high-efficacy DMT was estimated using logistic regression including sex, age at treatment initiation, calendar period of diagnosis, disease duration prior to treatment initiation, baseline EDSS, and MRI lesion burden (1–9, 10–20, > 20, and missing). Stabilized inverse probability weights were calculated and applied in weighted Cox proportional hazards models for RAW-CDW and PIRA-CDW. Follow-up definitions and censoring rules were identical to those in the primary analyses. This analysis was intended as a robustness assessment under an alternative adjustment framework and not as a formal causal model. All analyses were conducted in SAS 9.4 (SAS Institute, Cary, NC, USA).

## Results

We followed 2563 patients with relapsing-onset MS following DMT initiation. Mean duration between disease onset and DMT initiation was 3.4 years (SD 5.4), and mean age at DMT initiation was 37.2 years (SD 10.6). Baseline EDSS was higher among participants who initiated high-efficacy DMT (*p* < 0.0001), while there was no significant difference in disease duration at DMT initiation between those who initiated platform versus high-efficacy DMT. Baseline characteristics of participants are presented in Table 1.

**Table 1:** Characteristics of study population

Characteristic	Total	Initiating platform DMT	Initiating HE-DMT	*P* value
*N*		1987	576	
Age at disease onset, years (SD)	33.8 (10.1)	34.2 (10.1)	32.8 (10.0)	0.003
Time to diagnosis, years (SD)	3.0 (4.9)	3.1 (5.2)	2.5 (4.5)	< 0.0001
Age at time 0, years (SD)	37.2 (10.6)	37.6 (10.6)	36.0 (10.6)	0.002
Disease duration at time 0, years (SD)	3.4 (5.4)	3.4 (5.3)	3.2 (5.4)	0.44
Sex				
Female, *n* (%)	1826 (71.2)	1439 (72.4)	387 (67.2)	0.01
Male, *n* (%)	737 (28.8)	548 (27.6)	189 (32.8)	
Ancestry				
Nordic, *n* (%)	2039 (79.6)	1585 (79.8)	454 (78.8)	0.62
Non-Nordic, *n* (%)	524 (20.4)	402 (20.2)	122 (21.2)	
Pre-secondary, *n* (%)	268 (10.5)	212 (10.7)	56 (9.7)	0.57
Secondary, *n* (%)	1237 (48.3)	945 (47.6)	292 (50.7)	
Post-secondary, *n* (%)	1058 (41.3)	830 (41.8)	228 (39.6)	
EDSS at time 0, mean (SD)	1.6 (1.3)	1.5 (1.3)	2.0 (1.4)	< 0.0001
EDSS at time 0, median (IQR)	1.5 (1.0–2.5)	1.5 (1.0–2.0)	2.0 (1.0–3.0)	
MRI lesion burden within 12 months prior to DMT start, *n* (%)				
1–9	720 (28.1)	580 (29.2)	140 (24.3)	< 0.0001
10–20	443 (17.3)	331 (16.7)	112 (19.4)	
> 20	535 (20.9)	358 (18.0)	177 (30.7)	
Missing	865 (33.7)	718 (36.1)	147 (25.5)	
Mean follow-up, years (SD)	9.5 (4.3)	10.1 (4.1)	7.7 (3.7)	< 0.0001
Smoking				
Never, *n* (%)	1221 (47.6)	941 (47.4)	280 (48.6)	0.83
Current, *n* (%)	549 (21.4)	442 (22.2)	107 (18.6)	
Past, *n* (%)	883 (34.5)	604 (30.4)	189 (32.8)	
Snuff use, *n* (%)	470 (18.3)	367 (18.5)	103 (17.9)	0.73
Alcohol use, *n* (%)	1737 (67.8)	1380 (69.5)	357 (62.0)	0.0006
Past IM				
Yes, *n* (%)	475 (18.5)	356 (17.9)	119 (20.7)	0.002
No, *n* (%)	1790 (69.8)	1417 (71.3)	373 (64.8)	
Unsure, *n* (%)	298 (11.6)	214 (10.8)	84 (14.6)	
Body mass index (SD)	25.0 (5.1)	25.1 (5.2)	25.0 (4.9)	0.14
Obesity, *n* (%)	680 (26.6)	528 (26.6)	152 (26.4)	0.91
Low sun exposure, *n* (%)	956 (37.3)	720 (36.2)	236 (41.0)	0.04
Fish consumption score (SD)	3.9 (1.1)	3.9 (1.1)	3.9 (1.1)	0.72
Regular physical activity, *n* (%)	1003 (39.1)	757 (38.1)	246 (42.7)	0.05

DMT, disease-modifying therapy; HE, high-efficacy; EDSS, Expanded Disability Status Scale; SD, standard deviation; IM, infectious mononucleosis; time 0 represents DMT initiation

### DMT and time-to-event outcomes

At treatment initiation, 1987 participants started a platform DMT and 576 started a high-efficacy DMT. During follow-up, 542 participants experienced confirmed disability worsening, of whom 208 were classified as RAW and 334 as PIRA. Compared with the platform DMT group, the HR for RAW-CDW among those who initiated high-efficacy DMT was 0.60 (95% CI 0.38–0.92), and the HR for PIRA-CDW was 1.05 (95% CI 0.79–1.39) (Table 2).

**Table 2:** Sensitivity analysis with RAW-CDW redefined: risk of RAW-CDW and PIRA-CDW by initial treatment

	*N*	Person-years	Outcome (%)	HR (95% CI)^a^	HR (95% CI)^b^
RAW-CDW					
Platform DMT	1987	9653	182 (9.2)	1.0 (reference)	1.0 (reference)
HE-DMT	576	2898	26 (4.5)	0.48 (0.31–0.71)	0.60 (0.38–0.92)
PIRA-CDW					
Platform DMT	1987	9653	261 (13.1)	1.0 (reference)	1.0 (reference)
HE-DMT	576	2898	73 (12.8)	1.15 (0.88–1.49)	1.05 (0.79–1.39)

DMT, disease-modifying therapy; HE, high-efficacy; RAW-CDW, relapse-associated worsening leading to confirmed disability worsening; PIRA-CDW, progression independent of relapse activity leading to confirmed disability worsening; HR, hazard ratio

^a^Adjusted for sex and age at the treatment start

^b^Adjusted for sex, age at the treatment start, calendar period of diagnosis, baseline EDSS, and duration between onset and DMT start

In analyses of EDSS milestones, participants initiating high-efficacy DMT had a lower risk of reaching EDSS 3 (HR 0.26, 95% CI 0.17–0.38) and EDSS 4 (HR 0.32, 95% CI 0.18–0.54) (Table 3).

**Table 3:** Risk of reaching EDSS disability milestones by initial treatment

	*N*	Person-years	Outcome (%)	HR (95% CI)^a^	HR (95% CI)^b^
EDSS 3					
Platform DMT	1668	7471	300 (18.0)	1.0 (reference)	1.0 (reference)
HE-DMT	481	2363	29 (6.0)	0.32 (0.21–0.46)	0.26 (0.17–0.38)
EDSS 4					
Platform DMT	1668	7932	121 (7.3)	1.0 (reference)	1.0 (reference)
HE-DMT	481	2415	15 (3.1)	0.44 (0.25–0.73)	0.32 (0.18–0.54)

EDSS, expanded disability status scale; DMT, disease-modifying therapy; HE, high-efficacy; HR, hazard ratio

^a^Adjusted for sex and age at the treatment start

^b^Adjusted for sex, age at the treatment start, calendar period of diagnosis, baseline EDSS, and duration between disease onset and treatment start

### DMT and long-term disability trajectories

**Fig. 1:**
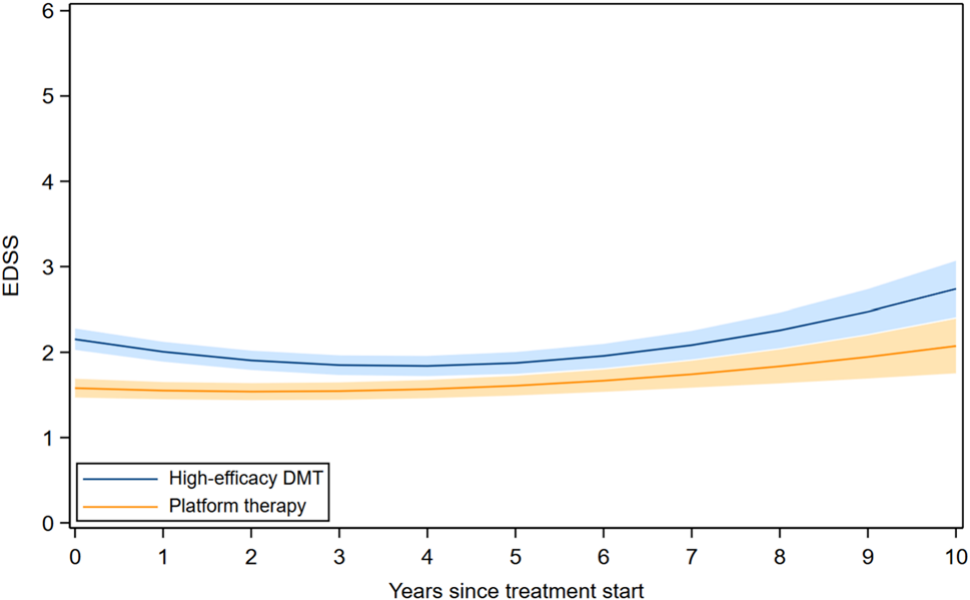
EDSS trajectories among DMT-naïve participants with relapsing-onset MS initiating platform versus high-efficacy DMT. EDSS, expanded disability status scale; DMT, disease-modifying therapy; MS, multiple sclerosis; HE, high-efficacy. Adjusted for age at treatment start, sex, calendar period of diagnosis, and disease duration at treatment start. The bands represent the 95% CI of estimated mean EDSS. *β* for initial treatment × time (HE-DMT vs. platform): − 0.05, 95% CI: − 0.07, − 0.02, *P*-value < 0.001; *β* for initial treatment × time^2^ (HE-DMT vs. platform): 0.01, 95% CI: 0.01, 0.02, *P*-value < 0.001

EDSS trajectories differed by initial treatment efficacy (Fig. 1). In the mixed-effects model, the interaction between initial treatment and time was negative (*β* = − 0.05, 95% CI − 0.07 to − 0.02), while the interaction with the quadratic time term was positive (*β* = 0.01, 95% CI 0.01–0.02), consistent with differences in early slope and subsequent curvature between groups. Participants initiating high-efficacy DMT started at higher mean EDSS and were relatively stable during the first years, followed by a more pronounced increase later in follow-up. Those starting on platform therapy began at lower EDSS but rose more steeply early on, resulting in partial convergence of the two trajectories over time.

**Fig. 2:**
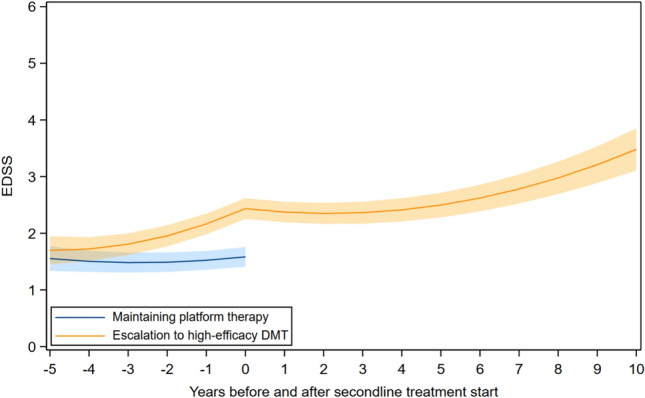
EDSS trajectory among participants with relapsing-onset MS escalating from platform to high-efficacy DMT. EDSS, expanded disability status scale; DMT, disease-modifying therapy; MS, multiple sclerosis; HE, high-efficacy; year 0, time of escalation. Adjusted for age at year 0, sex, calendar period of diagnosis, and disease duration at year 0. The bands represent the 95% CI of estimated mean EDSS. *β* for escalation × time before escalation (switch vs. non-switch): 0.23, 95% CI: 0.13, 0.33, *P*-value < 0.001; *β* for escalation × time^2^ before escalation (switch vs. non-switch): 0.02, 95% CI: − 0.004, 0.04, *P*-value = 0.12; *β* for time after escalation: − 0.26, 95% CI: − 0.33, − 0.19, *P*-value < 0.001; *β* for time^2^ after escalation: 0.02, 95% CI: 0.01, 0.02, *P*-value < 0.001

Figure 2 shows modeled EDSS trajectories among participants who either maintained platform DMT or escalated to high-efficacy DMT. Year 0 represents the time of escalation. Prior to escalation, individuals who later switched to high-efficacy DMT showed a steeper increase in EDSS compared with those who remained on platform DMT (*β* for escalation × time before escalation: 0.23, 95% CI 0.13–0.33). After escalation, the trajectory among switchers was initially flatter (*β* for time after escalation: − 0.26, 95% CI − 0.33 to − 0.19), followed by a gradual upward curvature later during follow-up (*β* for time^2^ after escalation: 0.02, 95% CI 0.01 to 0.02) (Fig. 2).

### Sensitivity analyses

In analyses restricted to participants with mild disability at treatment initiation (baseline EDSS < 3, *n* = 2111), the association between high-efficacy DMT and PIRA-CDW was not significant (HR 1.02, 95% CI 0.77–1.33), consistent with the primary analysis.

When RAW was alternatively defined as CDW events preceded by a clinical relapse within 90 days prior to the EDSS increase, the association with RAW-CDW was stronger (HR 0.16, 95% CI 0.03–0.56), while estimates for PIRA-CDW remained imprecise and compatible with no association (HR 0.97, 95% CI 0.75–1.22) (eTable 1).

Further adjustment of the primary analyses for additional demographic and lifestyle factors did not materially alter the effect estimates (eTable 2). Results also remained consistent when we restricted to participants diagnosed in 2010 or later (eTable 3), and when start of follow-up was redefined as the date of the baseline EDSS assessment rather than of DMT initiation (eTable 4).

To assess potential selection bias related to missing EDSS assessments during the initial DMT course, we compared participants excluded due to missing EDSS (*n* = 478) with those included in the primary analyses (*n* = 2563). Excluded individuals had shorter disease duration at DMT initiation (*p* = 0.003), were more often diagnosed in later calendar periods (*p* < 0.0001), and had lower educational level (*p* = 0.003). They more frequently initiated on platform DMTs (*p* < 0.0001) and more often escalated treatment during follow-up (*p* < 0.0001) (eTable 5). Of those who escalated, the median time before the switch was 0.81 years (IQR 0.33–1.68). When participants without EDSS assessments during the initial DMT course were included in a conservative sensitivity analysis assuming early treatment escalation reflected inflammatory disease activity, the adjusted HRs for RAW-CDW and PIRA-CDW remained similar to those observed in the primary analysis (eTable 6). The results from the IPW-weighted models were largely unchanged compared with the primary multivariable-adjusted analyses (eTable 7).

## Discussion

In this cohort of DMT-naïve individuals with relapsing-onset MS, initiation of a high-efficacy DMT was associated with a substantially lower risk of RAW-CDW, whereas we did not observe a significant association for PIRA-CDW compared to platform DMT. High-efficacy therapy was also associated with a lower HR of reaching EDSS milestones.

The dissociation between RAW and PIRA is clinically and biologically plausible. High-efficacy therapies primarily target focal inflammatory activity [18], consistent with the pronounced reduction in RAW-CDW. In contrast, we did not observe a significant association between initial treatment efficacy and PIRA-CDW. The point estimate was close to unity and did not suggest a directional trend. This pattern is compatible with non-relapse progression reflecting slowly expanding lesions, diffuse microglial activation, and neurodegenerative processes that may be only partially modifiable by anti-inflammatory mechanisms [4–6].

Our findings are broadly consistent with earlier observational studies demonstrating short-term disability benefits of high-efficacy therapy [10–13], although most prior studies did not explicitly separate RAW from PIRA, limiting direct comparability. In a recent study of late-onset MS that separated these components, no clear association was observed between higher efficacy therapy and relapse-independent progression, a pattern similar to that observed in our study [19]. The overall pattern also aligns with recent Swedish real-world data, where structured monitoring and timely escalation reduce time spent on insufficiently effective therapy and may attenuate long-term contrasts in EDSS-based outcomes [14]. Since EDSS milestones capture cumulative disability over longer periods of time, they may be more sensitive to the downstream benefits of relapse suppression. This pattern is consistent with the possibility that relapse-independent progression is driven by biological mechanisms less responsive to anti-inflammatory therapy, and that the structured treatment context with active monitoring and timely escalation, may attenuate observable differences between efficacy classes.

The modeled EDSS trajectories suggest partial convergence without crossover, implying that early between-group differences narrow over time but do not reverse, consistent with stronger early inflammatory control under high-efficacy therapy, rather than a sustained effect on relapse-independent progression.

The same temporal dynamics were evident in the analysis of treatment escalation. Future switchers accumulated disability more rapidly before escalation and had higher EDSS at the time of switch than non-switchers. After escalation, their trajectory initially flattened and then gradually increased, consistent with escalation triggered by breakthrough inflammatory activity and providing a pragmatic view of real-world treatment decisions and subsequent disability course.

Sensitivity analyses supported the stability of the primary findings. Consistent results were observed when analyses were restricted to participants with mild baseline disability, indicating that the absence of a detectable association with PIRA-CDW was not driven by disease stage at treatment initiation. In analyses using an alternative, narrower relapse ascertainment window, the association with RAW-CDW was stronger, while estimates for PIRA-CDW remained compatible with no association. The difference in effect size between the primary and 90-day relapse definitions likely reflects differences in sensitivity and specificity of RAW classification. A broader window increases sensitivity for capturing relapse-associated disability but may include events temporally distant from the EDSS increase, whereas a narrower window increases specificity at the expense of reduced event numbers. The consistent direction of association across definitions supports the robustness of the inflammatory contrast.

Since early platform switchers are typically escalated due to inflammatory breakthrough, their exclusion preferentially removes relapse-driven worsening from the platform group and inflates the relative contribution of PIRA. When these individuals were reintroduced under a conservative assumption that early escalation reflected inflammatory disease activity, platform RAW increased as expected, but the null association between high-efficacy therapy and PIRA-CDW persisted. Results were likewise unchanged when follow-up was re-anchored at the first EDSS assessment to eliminate potential immortal time. Together, these analyses reduce the likelihood that the null association with PIRA-CDW is fully attributable to selective exclusion, timing-related bias, or the specific relapse ascertainment period used to define RAW.

From a clinical perspective, our findings indicate that high-efficacy DMT is associated with lower risk of relapse-driven worsening. While relapse-independent progression measured as PIRA-CDW was not significantly reduced, the risk of reaching EDSS milestones over follow-up was lower with high-efficacy DMT. These results support continued emphasis on aggressive control of inflammatory activity and prompt reassessment of treatment strategy when relapse activity or early disability accumulation occurs. They also point to the need for complementary approaches that target neuroprotection and repair to slow non-relapse progression.

The study’s longitudinal design with long-term follow-up enabled characterization of disability accumulation over time, and the use of real-world clinical data enhances the generalizability of the findings to everyday clinical practice. Classifying exposure at the efficacy class level simplifies interpretation when within-class switching is common, but it prevents evaluation of individual substances and may mask within-class heterogeneity.

We analyzed RAW and PIRA as distinct endpoints, which clarifies effects along inflammatory and non-inflammatory axes. Definitions of RAW and PIRA vary across studies, particularly between randomized trials with protocol-driven EDSS confirmation and registry-based follow-up with variable visit intervals. Our applied definition was designed to reflect routine clinical practice, and sensitivity analyses applying narrower a 90-day relapse window yielded consistent directional results. Nevertheless, outcome classification remains dependent on documentation and confirmation windows. Incomplete or delayed relapse reporting could influence the classification of disability worsening events. In addition, clinically defined PIRA does not exclude concurrent subclinical inflammatory activity detectable only on MRI, indicating that the distinction between relapse-associated and relapse-independent worsening is partly operational rather than purely biological. While differential relapse capture between treatment groups cannot be entirely excluded, the substantially lower risk of RAW observed among participants initiating high-efficacy DMT and the stability of findings across sensitivity analyses suggest that misclassification alone is unlikely to account for the overall pattern of associations. Although the point estimate for the association between initiation of high-efficacy DMT and the risk of PIRA was close to unity and did not suggest a directional trend, the confidence interval allows for moderate deviations from the null, and smaller effect sizes cannot be ruled out given the observed precision.

Treatment decisions in routine clinical practice are influenced by physician perception of disease aggressiveness, prior inflammatory activity, and MRI findings. Although we adjusted for key covariates and conducted a propensity score-weighted sensitivity analysis, residual confounding by indication cannot be excluded. Notably, despite this potential bias, initiation of high-efficacy DMT remained associated with a substantially lower risk of RAW and delayed attainment of EDSS milestones. Since follow-up was censored at treatment switch or discontinuation, informative censoring related to early disease activity or treatment response cannot be excluded. Although we conducted sensitivity analyses to explore the potential impact of early switching, time-dependent treatment changes and time-varying confounding were not explicitly modeled, and the present results should therefore be interpreted as associations under sustained initial treatment exposure. EDSS, being ordinal and non-linear, was modeled as continuous in mixed-effects models with quadratic time terms, which imposes parametric assumptions regarding functional form. These trajectory analyses should therefore be interpreted as descriptive summaries. Finally, information on specific clinical manifestations at disease onset was not consistently available and could therefore not be included in the analyses.

In summary, initiation of high-efficacy DMT was associated with a substantially lower risk of RAW-CDW and with lower risk of reaching EDSS milestones, whereas no significant association was observed for PIRA-CDW in etiologic models over the available follow-up. These findings suggest differential associations with inflammatory and relapse-independent components of MS disability, emphasizing the need of complementary strategies aimed at neuroprotection and repair.

## Supplementary Information

Below is the link to the electronic supplementary material.

Supplementary file1 (DOCX 31 KB)

## Data Availability

Anonymized data underlying this article will be shared on reasonable request from any qualified investigator that wants to analyze questions that are related to the published article.
