# Supplementary material for: Long-term disability after initiation of platform versus high-efficacy disease-modifying therapy in relapsing-onset multiple sclerosis
Source: J Neurol. 2026 Apr 1;273(4):240. doi: 10.1007/s00415-026-13790-5 (PMC13043616; doi:10.1007/s00415-026-13790-5)
Supplement: Supplementary file 1 — Supplementary file1 (DOCX 31 KB) [file 415_2026_13790_MOESM1_ESM.docx]

**Supplementary data**

eTable 1. Sensitivity analysis with RAW-CDW redefined: Risk of RAW-CDW and PIRA-CDW by initial treatment.

eTable 2. Sensitivity analysis including additional demographic and lifestyle covariates: Risk of RAW-CDW and PIRA-CDW by initial treatment.

eTable 3. Sensitivity analysis limited to participants diagnosed in 2010 or later: Risk of RAW-CDW and PIRA-CDW by initial treatment.

eTable 4. Sensitivity analysis with follow-up time redefined: Risk of RAW-CDW and PIRA-CDW by initial treatment.

eTable 5. Comparison of participants with and without EDSS follow-up during the initial DMT course.

eTable 6. Sensitivity analysis including participants without EDSS: Risk of RAW-CDW and PIRA-CDW by initial treatment.

eTable 7. Inverse probability-weighted Cox models for RAW-CDW and PIRA-CDW by initial treatment.

eTable 1. Sensitivity analysis with RAW-CDW redefined: Risk of RAW-CDW and PIRA-CDW by initial treatment.

| RAW-CDW | | | | | |
| --- | --- | --- | --- | --- | --- |
|  | N | Person-years | Outcome (%) | HR (95% CI)^1^ | HR (95% CI)^2^ |
| Platform DMT | 1987 | 9653 | 49 (2.5) | 1.0 (reference) | 1.0 (reference) |
| HE-DMT | 576 | 2898 | 2 (0.4) | 0.12 (0.02-0.39) | 0.16 (0.03-0.56) |
| PIRA-CDW | | | | | |
|  | N | Person-years | Outcome (%) | HR (95% CI)^1^ | HR (95% CI)^2^ |
| Platform DMT | 1987 | 9653 | 394 (19.8) | 1.0 (reference) | 1.0 (reference) |
| HE-DMT | 576 | 2898 | 97 (16.8) | 0.96 (0.77-1.20) | 0.96 (0.75-1.22) |

DMT=disease-modifying therapy; HE=high-efficacy; RAW-CDW=relapse-associated worsening leading to confirmed disability worsening; PIRA-CDW=progression independent of relapse activity leading to confirmed disability worsening; HR=hazard ratio; CDW events were classified as RAW in the case of relapse occurrence within 90 days prior to the EDSS increase; CDW events not meeting the relapse criteria were classified as PIRA. ^1^adjusted for sex and age at the treatment start; ^2^adjusted for sex, age at the treatment start, calendar period of diagnosis, baseline EDSS, and duration between onset and DMT start.

eTable 2. Sensitivity analysis including additional demographic and lifestyle covariates: Risk of RAW-CDW and PIRA-CDW by initial treatment.

| RAW-CDW | | | | | |
| --- | --- | --- | --- | --- | --- |
|  | N | Person-years | Outcome (%) | HR (95% CI)^1^ | HR (95% CI)^2^ |
| Platform DMT | 1987 | 4.9 (3.6) | 49 (2.5) | 1.0 (reference) | 1.0 (reference) |
| HE-DMT | 576 | 5.0 (2.9) | 2 (0.4) | 0.12 (0.02-0.39) | 0.14 (0.03-0.59) |
| PIRA-CDW | | | | | |
|  | N | Person-years | Outcome (%) | HR (95% CI)^1^ | HR (95% CI)^2^ |
| Platform DMT | 1987 | 4.9 (3.6) | 394 (19.8) | 1.0 (reference) | 1.0 (reference) |
| HE-DMT | 576 | 5.0 (2.9) | 97 (16.8) | 0.96 (0.77-1.20) | 1.01 (0.80-1.29) |

DMT=disease-modifying therapy; HE=high-efficacy; RAW-CDW=relapse-associated worsening leading to confirmed disability worsening; PIRA-CDW=progression independent of relapse activity leading to confirmed disability worsening; HR=hazard ratio; ^1^adjusted for sex and age at the treatment start; ^2^adjusted for sex, age at the treatment start, ancestry, calendar period of diagnosis, duration between onset and DMT start, baseline EDSS, educational attainment, another autoimmune disease, past infectious mononucleosis, smoking, alcohol use, body mass index, fish consumption, sun exposure habits, and physical activity.

eTable 3. Sensitivity analysis limited to participants diagnosed in 2010 or later: Risk of RAW-CDW and PIRA-CDW by initial treatment.

| RAW-CDW | | | | | |
| --- | --- | --- | --- | --- | --- |
|  | N | Person-years | Outcome (%) | HR (95% CI)^1^ | HR (95% CI)^2^ |
| Platform DMT | 1027 | 3792 | 15 (1.5) | 1.0 (reference) | 1.0 (reference) |
| HE-DMT | 463 | 2137 | 1 (0.2) | 0.11 (0.01-0.83) | 0.10 (0.01-0.79) |
| PIRA-CDW | | | | | |
|  | N | Person-years | Outcome (%) | HR (95% CI)^1^ | HR (95% CI)^2^ |
| Platform DMT | 1027 | 3792 | 152 (14.8) | 1.0 (reference) | 1.0 (reference) |
| HE-DMT | 463 | 2137 | 63 (13.6) | 0.82 (0.60-1.11) | 0.88 (0.63-1.18) |

DMT=disease-modifying therapy; HE=high-efficacy; RAW-CDW=relapse-associated worsening leading to confirmed disability worsening; PIRA-CDW=progression independent of relapse activity leading to confirmed disability worsening; HR=hazard ratio; ^1^adjusted for sex and age at the treatment start; ^2^adjusted for sex, age at the treatment start, baseline EDSS, and duration between onset and DMT start.

eTable 4. Sensitivity analysis with follow-up time redefined: Risk of RAW-CDW and PIRA-CDW by initial treatment.

| RAW-CDW | | | | | |
| --- | --- | --- | --- | --- | --- |
|  | N | Person-years | Outcome (%) | HR (95% CI)^1^ | HR (95% CI)^2^ |
| Platform DMT | 1949 | 8661 | 49 (2.5) | 1.0 (reference) | 1.0 (reference) |
| HE-DMT | 568 | 2758 | 2 (0.4) | 0.11 (0.02-0.37) | 0.16 (0.02-0.53) |
| PIRA-CDW | | | | | |
|  | N | Person-years | Outcome (%) | HR (95% CI)^1^ | HR (95% CI)^2^ |
| Platform DMT | 1949 | 8661 | 394 (20.2) | 1.0 (reference) | 1.0 (reference) |
| HE-DMT | 568 | 2758 | 97 (17.1) | 0.87 (0.69-1.08) | 0.88 (0.69-1.11) |

DMT=disease-modifying therapy; HE=high-efficacy; RAW-CDW=relapse-associated worsening leading to confirmed disability worsening; PIRA-CDW=progression independent of relapse activity leading to confirmed disability worsening; HR=hazard ratio. Follow-up was calculated from baseline EDSS. ^1^adjusted for sex and age at the treatment start; ^2^adjusted for sex, age at the treatment start, calendar period of diagnosis, baseline EDSS, and duration between onset and DMT start.

eTable 5. Comparison of participants with and without EDSS follow-up during the initial DMT course.

| Characteristic | | | Study sample | Excluded | P value |
| --- | --- | --- | --- | --- | --- |
| N | | | 2563 | 478 |  |
| Age at disease onset, years (SD) | | | 33.8 (10.1) | 33.9 (10.6) | 0.90 |
| Calendar period of diagnosis | | <2005 | 188 (7.3) | 8 (1.7) | <0.0001 |
|  |  | 2005-2009 | 885 (34.5) | 152 (31.8) |  |
|  |  | 2010- | 1490 (58.1) | 318 (66.5) |  |
| Age at time 0, years (SD) | | | 37.2 (10.6) | 36.5 (10.8) | 0.19 |
| Disease duration at time 0, years (SD) | | | 3.4 (5.4) | 2.6 (4.3) | 0.003 |
| Sex | Female, n (%) | | 1826 (71.2) | 344 (72.0) | 0.75 |
|  | Male, n (%) | | 737 (28.8) | 134 (28.0) |  |
| Ancestry | Nordic, n (%) | | 2039 (79.6) | 393 (82.2) | 0.18 |
|  | Non-Nordic, n (%) | | 524 (20.4) | 85 (17.8) |  |
| Education | Pre-secondary, n (%) | | 268 (10.5) | 67 (14.0) | 0.003 |
|  | Secondary, n (%) | | 1237 (48.3) | 251 (52.5) |  |
|  | Post-secondary, n (%) | | 1058 (41.3) | 160 (33.5) |  |
| First recorded EDSS, mean (SD)  First recorded EDSS, median (IQR) | | | 1.6 (1.3) 1.5 (1.0-2.5) | 2.0 (1.6) 2.0 (1.0-3.0) | <0.0001 |
| Initial DMT | Platform DMT, n (%) | | 1987 (77.5) | 424 (92.0) | <0.0001 |
|  | HE-DMT, n (%) | | 576 (22.5) | 54 (8.0) |  |
| Escalation during follow-up, n (%) | | | 929 (36.3) | 288 (60.3) | <0.0001 |
| Smoking | Never, n (%) | | 1221 (47.6) | 213 (44.6) | 0.48 |
|  | Current, n (%) | | 549 (21.4) | 121 (25.3) |  |
|  | Past, n (%) | | 883 (34.5) | 144 (30.1) |  |
| Snuff use, n (%) | | | 470 (18.3) | 71 (13.1) | 0.07 |
| Alcohol use, n (%) | | | 1737 (67.8) | 292 (61.1) | 0.003 |
| Past IM | Yes, n (%) | | 475 (18.5) | 98 (20.5) | 0.10 |
|  | No, n (%) | | 1790 (69.8) | 316 (66.1) |  |
|  | Unsure, n (%) | | 298 (11.6) | 64 (13.4) |  |
| Body mass index (SD) | | | 25.1 (5.1) | 25.1 (5.2) | 0.31 |
| Obesity, n (%) | | | 680 (26.6) | 139 (29.1) | 0.26 |
| Low sun exposure, n (%) | | | 956 (37.3) | 195 (40.8) | 0.15 |
| Fish consumption score (SD) | | | 3.9 (1.1) | 3.8 (1.1) | 0.67 |
| Regular physical activity, n (%) | | | 1003 (39.1) | 160 (33.5) | 0.02 |

DMT=disease-modifying therapy; HE=high-efficacy; EDSS=Expanded Disability Status Scale; SD=standard deviation; IQR=interquartile range; IM=infectious mononucleosis; Time 0 represents DMT initiation.

eTable 6. Sensitivity analysis including participants without EDSS: Risk of RAW-CDW and PIRA-CDW by initial treatment.

| RAW-CDW | | | | | |
| --- | --- | --- | --- | --- | --- |
|  | N | Person-years | Outcome (%) | HR (95% CI)^1^ | HR (95% CI)^2^ |
| Platform DMT | 2420 | 30110 | 429 (17.7) | 1.0 (reference) | 1.0 (reference) |
| HE-DMT | 621 | 2939 | 2 (0.3) | 0.18 (0.03-0.59) | 0.16 (0.03-0.56) |
| PIRA-CDW | | | | | |
|  | N | Person-years | Outcome (%) | HR (95% CI)^1^ | HR (95% CI)^2^ |
| Platform DMT | 2420 | 30110 | 394 (16.3) | 1.0 (reference) | 1.0 (reference) |
| HE-DMT | 621 | 2939 | 97 (15.6) | 1.45 (1.15-1.81) | 0.96 (0.75-1.22) |

DMT=disease-modifying therapy; HE=high-efficacy; RAW-CDW=relapse-associated worsening leading to confirmed disability worsening; PIRA-CDW=progression independent of relapse activity leading to confirmed disability worsening; HR=hazard ratio. Handling of participants without an EDSS assessment: platform initiators who escalated to HE were assigned an imputed RAW-CDW event at switch – 90 days, and follow-up accrued from DMT initiation until switch - 90 days. Those who remained in the same DMT class >365 days were right-censored at day 365, and those treated <365 days were right-censored at treatment stop. ^1^adjusted for sex and age at the treatment start; ^2^adjusted for sex, age at the treatment start, calendar period of diagnosis, baseline EDSS, and duration between onset and DMT start.

eTable 7. Inverse probability-weighted Cox models for RAW-CDW and PIRA-CDW by initial treatment.

| RAW-CDW | | | | |
| --- | --- | --- | --- | --- |
|  | N | Person-years | Outcome (%) | IPW-HR (95% CI) |
| Platform DMT | 1987 | 9653 | 182 (9.2) | 1.0 (reference) |
| HE-DMT | 576 | 2898 | 26 (4.5) | 0.56 (0.40-0.89) |
| PIRA-CDW | | | | |
|  | N | Person-years | Outcome (%) | IPW-HR (95% CI) |
| Platform DMT | 1987 | 9653 | 261 (13.1) | 1.0 (reference) |
| HE-DMT | 576 | 2898 | 73 (12.8) | 1.04 (0.85-1.39) |

DMT=disease-modifying therapy; HE=high-efficacy; RAW-CDW=relapse-associated worsening leading to confirmed disability worsening; PIRA-CDW=progression independent of relapse activity leading to confirmed disability worsening; HR=hazard ratio; IPW=inverse probability of treatment weighting. Models were weighted using stabilized inverse probability weights derived from a propensity score including sex, age at treatment start, calendar period of diagnosis, baseline EDSS, MRI lesion burden, and disease duration between onset and DMT start. Follow-up definitions and censoring rules were identical to the primary analyses.
